# Investigation of Specific Targeting of Triptorelin-Conjugated Dextran-Coated Magnetite Nanoparticles as a Targeted Probe in GnRH^+^ Cancer Cells in MRI

**DOI:** 10.1155/2021/5534848

**Published:** 2021-05-17

**Authors:** Milad Yousefvand, Zahra Mohammadi, Farzaneh Ghorbani, Rasoul Irajirad, Hormoz Abedi, Somayyeh Seyedi, Arash Papi, Alireza Montazerabadi

**Affiliations:** ^1^Medical Physics Research Center, Mashhad University of Medical Sciences, Mashhad, Iran; ^2^Radiological Technology Department of Actually Paramedical Sciences, Babol University of Medical Sciences, Babol, Iran; ^3^Student Research Committee, Mashhad University of Medical Sciences, Mashhad, Iran; ^4^Finetech in Medicine Research Center, Iran University of Medical Sciences, Tehran, Iran

## Abstract

In recent years, the conjugation of superparamagnetic iron oxide nanoparticles (SPIONs), as tumor-imaging probes for magnetic resonance imaging (MRI), with tumor targeting peptides possesses promising advantages for specific delivery of MRI agents. The objective of the current study was to design a targeted contrast agent for MRI based on Fe_3_O_4_ nanoparticles conjugated triptorelin (SPION@triptorelin), which has a great affinity to the GnRH receptors. The SPIONs-coated carboxymethyl dextran (SPION@CMD) conjugated triptorelin (SPION@CMD@triptorelin) were synthesized using coprecipitation method and characterized by DLS, TEM, XRD, FTIR, Zeta, and VSM techniques. The relaxivities of synthetized formulations were then calculated using a 1.5 Tesla clinical magnetic field. MRI, quantitative cellular uptake, and cytotoxicity level of them were estimated. The characterization results confirmed that the formation of SPION@CMD@triptorelin has been conjugated with a suitable size. Our results demonstrated the lack of cellular cytotoxicity of SPION@CMD@triptorelin, and it could increase the cellular uptake of SPIONs to MDA-MB-231 cancer cells 6.50-fold greater than to SPION@CMD at the concentration of 75 *μ*M. The relaxivity calculations for SPION@CMD@triptorelin showed a suitable *r*_2_ and *r*_2_/*r*_1_ with values of 31.75 mM^−1^·s^−1^ and 10.26, respectively. Our findings confirm that triptorelin-targeted SPIONs could provide a *T*_2_-weighted probe contrast agent that has the great potential for the diagnosis of GnRH-positive cancer in MRI.

## 1. Introduction

Cancers are one of the most frequent mortality causes worldwide due to the challenges arising in the diagnosis and clinical management of the cancerous patients [1, 2]. However, conventional imaging techniques have played an effective role in the diagnosis of diseases, but they suffer from low specificity. Targeted nanomolecular imaging has been proposed as a suitable solution for early detection and improvement of contrast-to-noise ratio. Therefore, the developments of novel efficient approaches for early diagnosis of cancer are of paramount importance for decreasing of cancer mortality [3]. Moreover, the detection of cancer based on specific biomarkers or receptors has resulted in substantial improvements in early and specific diagnosis of human cancers, as well as monitoring the outcome of the disease during and after the course of the treatment [4–6].

Several methods (including physical, laboratory, biopsy, and imaging tests) are used for the diagnosis of various human cancers. Imaging tests as a noninvasive way allow examining bones and internal organs. The most common imaging methods that are used in diagnosing cancer may include a computerized tomography (CT) scan, bone scan, ultrasound, X-ray, positron emission tomography (PET) scan, and magnetic resonance imaging (MRI) [7, 8]. Over the last few years, researchers have used a range of selective and sensitive contrast agents for specific diagnosis of cancers by MRI in early stage.

The superparamagnetic iron oxide nanoparticles (SPIONs) are one of the US Food and Drug Administration- (FDA-) approved nanoparticles that are successfully used as tumor imaging probes for MRI [9, 10]. They can be also used for drug delivery due to their low toxicity, biocompatibility, and biodegradability, demonstrating a great potential for theranostic applications [10–14]. The coating of SPIONs with organic materials can also improve the colloidal stability, which can facilitate the implementation of SPIONs as contrast agents for MRI [15, 16]. Conjugation of SPIONs with tumor targeting moieties such as antibodies, aptamers, or peptides represents a promising platform for selective diagnosis of cancer biomarker/receptors and specific delivery of MRI agents. The specific binding of a peptide to its receptors (specific ligand-receptor interaction) which are overexpressed in cancer cells, resulting in efficient internalization of SPIONs into the cells based on receptor-mediated endocytosis (RME) [17, 18]. Moreover, it possibly helps a delay in extravasations from tumor tissues, thereby increasing the residence time of MRI agents in the tumor site.

Triptorelin is a synthetic decapeptide gonadotropin-releasing hormone (GnRH) agonist with similar structure to native GnRH and a great affinity to the GnRH receptors. Triptorelin is a potent inhibitor of the synthesis of testosterone (in men) and estrogen (in women) and is utilized to treat advanced prostate cancer and breast cancer due to the downregulation of cancer cell proliferation [19, 20].

**Figure 1:**
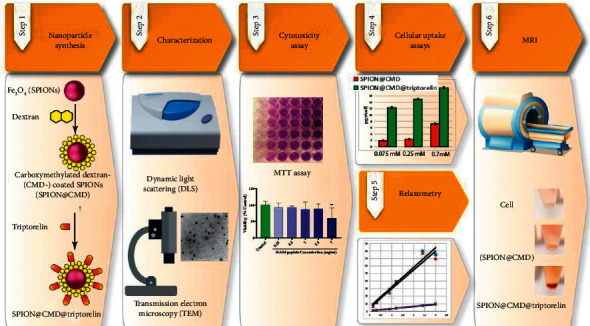
Schematic illustration of triptorelin-coated carboxymethylated dextran-coated SPIONs (SPION@CMD@triptorelin) as a targeting system for MRI.

The conjugation of dextran-coated SPIONs with triptorelin, as a targeting molecule, can provide a proper MRI probe for the tumor diagnosis [21]. Therefore, in this study, the triptorelin peptide was conjugated to synthesized SPION@CMD. Then, the morphological properties and size dispersity of the prepared nanoparticles were assessed using transmission electron microscope (TEM) and dynamic light scattering (DLS). In addition, the cytotoxicity of formulations was also investigated. In order to investigate the potential of synthesized SPION@CMD@triptorelin as a diagnostic nanoprobe, the MRI technique was carried out in vitro on MDA-MB-231 as GnRH-positive breast cancer cell line. Also, quantitative cellular uptake of the nanoprobe was obtained with atomic absorption spectroscopy technique. Work flow of this study has been shown in Figure 1.

## 2. Material and Methods

### 2.1. Materials

Dextran, iron(II) chloride tetrahydrate (FeCl_2_·4H_2_O, 99%), iron(III) chloride hexahydrate (FeCl_3_·6H_2_O, 99%), NaOH, bromoacetic acid, ammonium hydroxide (5 M), and 2,5-diphenyltetrazolium bromide (MTT) were purchased from Sigma-Aldrich (Munich, Germany). N50 neodymium magnet (50 × 50 × 30 mm) with 14 kilo Gauss remanence was purchased from Kaiven, Inc. (China). All materials were of analytical grade. Cell culture media (RPMI1640), fetal bovine sera (FBS), trypsin, and penicillin/streptomycin solution were obtained from Gibco (Darmstadt, Germany). MDA-MB-231 cell line was obtained from the National Cell Bank of Iran, Pasteur Institute of Iran.

### 2.2. Preparation of CMD-Coated SPIONs

Carboxymethylated dextran- (CMD-) coated superparamagnetic iron oxide nanoparticles (SPIONs) were synthesized using a coprecipitation method in an alkaline aqueous environment under nitrogen (N_2_) atmosphere. Briefly, 1 g dextran was dissolved in 3 mL deionized water; then, 3 ml of 8 M NaOH solution that also contained 0.1 M bromoacetic acid was added. The reaction mixture was heated to 60–65°C for 120 min and CMD was deposited with ethanol and was dried at 50°C in dry heat. Next, 25 mL of this aqueous solution containing 1 g of CMD was added to an aqueous solution (10 mL in deionized water) of 200 mg FeCl_2_·4H_2_O and 550 mg FeCl_3_·6H_2_O in the molar ratio 2 : 1 in deoxygenated deionized water with N_2_ and was stirred. 2.5 mL of 28% NH_4_OH was added slowly to this solution while stirred under N_2_ atmosphere at 10°C until the color of reaction turned to a deep black. Then, the suspension was heated up to 78°C for 1 h. The reaction mixture was cooled to the room temperature and centrifuged at 3500 rpm to remove very large aggregates. Afterwards, the SPION@CMD product was separated by applying an external magnet and washed with double-distilled water (ddH2O) twice, and after freeze-drying, lyophilized SPION@CMD was obtained.

### 2.3. Conjugation of Triptorelin to SPION@CMD

For the conjugation of triptorelin to SPION@CMD, the carboxyl-modified SPION@CMD was linked covalently to amine of triptorelin peptide using the isourea bonds coupling reaction. Briefly, 4.6 mg cyanogen bromide (BrCN) was added to an alkaline suspension (pH 10) of 5 g/mL SPION@CMD and 0.0211 g/mL sodium carbonate (Na_2_CO_3_) and was gently shaken for 1 h at room temperature to activate the terminal carboxyl group on SPION@CMD. Then, pH of the reaction mixture was reduced to 8.5 with the addition of 0.0238 g sodium dihydrogen phosphate (NaH_2_PO_4_), and then 4.8 mg triptorelin peptide was supplemented to the reaction medium and stirred for 2 h at room temperature. In the next step, the mixture was stirred in the presence of 3.2 mg glycine in 4°C for 24 hours. The formed conjugations (SPION@CMD@triptorelin) were collected using dialysis method by a membrane bag with a 20,000 cut-off molecular weight for 24 h and the excess reactants were removed.

### 2.4. Physicochemical Characterizations

The particle size distributions of SPION@CMD and SPION@CMD@triptorelin were measured by DLS using a HORIBA Zetasizer (NANO-ZS, Malvern, UK). The morphology of nanoparticles was carried out with transmission electron microscopy (TEM) (Philips CM120, Philips Electron Optics, the Netherlands). Crystal lattice structure of SPION@CMD was indicated by the X-ray diffraction (XRD) (GNR EXPLORER, Italy) at the room temperature. The XRD system (X-ray diffractometer) was run at 40 kV and 30 mA in a 2*θ* range of 20°–80°. The functional groups and chemical structural changes in SPION@CMD and SPION@triptorelin were obtained using Fourier transform infrared spectroscopy (FTIR). A vibrating sample magnetometer (VSM) (Danesh Pajoush Magnetis Company of Kashan, VSMF model, Iran) was used to evaluate the magnetic field-dependent magnetization under circulate magnetic field in the range of −15000 up to 15000 Oe at room temperature.

### 2.5. Cellular Uptake Level of Synthesized Formulations

The cellular uptake levels of synthesized formulations were assessed by the estimation of intracellular iron which was introduced to cells using SPION@CMD and SPION@CMD@triptorelin. The MDA-MB-231 cancer cells were seeded in 6-well plates at the density of 4 × 105 cells/well and incubated overnight. Then, the cells were washed and incubated with 0.075, 0.25, and 0.7 mM of SPION@CMD and SPION@CMD@triptorelin for 24 h. Then, the cells were washed three times with PBS and break down with perchloric acid. Subsequently, the concentration of Fe in cells was estimated with atomic absorption spectroscopy (AAS).

### 2.6. Cell Viability Assay of the Synthesized Formulations

MDA-MB-231 cells were seeded at a density of 8 × 103 per well in 96-well plates and incubated overnight at 37°C with 5% CO_2_ in air. 0.025, 0.05, 0.1, 0.25, and 0.7 mM of SPION@CMD and SPION@CMD@triptorelin were separately added to the culture medium and incubation was sustained for an additional 24 h. Then, the culture media were replaced with fresh medium, and finally, the MTT assay was performed after 48 h. After incubation of 20 *μ*L/well of MTT solution (5 mg/mL in PBS) for 4 h, the medium of each well was completely removed and 100 *μ*L of DMSO was added to dissolve crystals of formazan at room temperature. The absorbance was measured at a wavelength of 545 nm with a reference wavelength of 630 nm using an ELISA reader (Stat Fax-2100 Awareness, USA).

### 2.7. Relaxometry

The MR capability of SPION@CMD and SPION@CMD@triptorelin was carried out based on the relaxation rates (**R**=(1/**T**_1,2_)) which increase linearly with the SPIONs concentration according to the following equation:

(1) $$\begin{array}{c} \frac{1}{T_{1,2}}=\frac{1}{T_{0}}+r_{1,2}C, \end{array}$$

where 1/*T*_0_ is the relaxation rate of pure water and *C* is the concentration of SPIONs.

Longitudinal and transversal relaxivities' values (*r*_1_ and *r*_2_) were measured at 1.5 Tesla MRI scanner (Avanto/SIEMENS, Kamyab Hospital), using a phantom containing SPION@CMD@triptorelin with various concentrations of 0.15, 0.30, 0.9, 1.20, 2.40, and 3.0 mM.

T1-weighted images were acquired at time of echo (TE): 8.7 ms; time of repetition: TR1 to TR6: 100/300/600/ 900/1200/2000 ms; flip angle: 20 degree; matrix size: 256 × 192; field of view (FoV): 260 mm; 100%; averages: 1, echo train length: 1; and slice thickness: 5 mm. *T*_2_-weighted images were obtained with a *T*_2_ spin echo multisection pulse sequence by fixed TR of 2000 ms; TE1 to TE16: 13.8/27.9/41.4/55.2/69.0/82.8/96.6/110.4/124.2/138.0/151.8/165.6/179.4/193.2/207.0/220.8 ms; flip angle: 20 degree; matrix size: 256 × 192; FoV: 260 mm; 100%; averages: 1; and echo train length: 1. All curve fitting routines, which were used to determine the relaxation rate maps, were performed by Origin, Excel, and RadiAnt DICOM Viewer software.

### 2.8. Magnetic Resonance Imaging

MRI technique was used to visualize the accumulation of prepared SPION@CMD and SPION@CMD@triptorelin in MDA-MB-231 cells. MDA-MB-231 cells (1.5 × 106) were seeded in three single T25 flasks and incubated overnight at 37°C. Following incubation, SPION@CMD and SPION@CMD@triptorelin with concentrations of 0.25 mM were added to each T25 flask and incubated for 24 h. Then, cells were washed with PBS, and 3 × 106 cells were placed in 2 mL tubes in 0.4% agarose solution. Cells in agarose without incubation with nanoparticles were used as the control. The *T*_2_-weighted images were obtained using a 1.5 T MRI scanner with the following parameters: TR/TE: 2197/110 ms; flip angle: 180°; slice thickness: 3.0 mm; interslice distance: 1.2 mm; FoV: 1.2 × 1.2 cm; and matrix size: 128 × 128.

### 2.9. Statistical Analysis

SPSS 22 was used for data analysis. The nonparametric Kolmogorov–Smirnov test was used to assess the normality of data to serve as a goodness of fit test. One-way ANOVA followed by Tukey's multiple comparison tests were used to analyze statistical differences (*P* < 0.05) for a comparison of all groups.

## 3. Results

### 3.1. Physicochemical Characterizations of Nanoparticles

**Figure 2:**
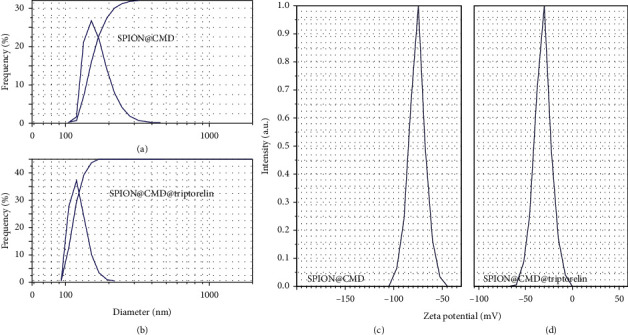
The particle size distribution diagram of (a) SPION@CMD and (b) SPION@CMD@triptorelin peaks at 160 nm and 116 nm, respectively. Zeta potential diagrams of (c) SPION@CMD and (d) SPION@CMD@triptorelin were measured at ∼−72.4 mV and ∼−31.5 mV, respectively.

Regarding the DLS results, only one peak was observed in both SPION@CMD and SPION@CMD@triptorelin (Figures 2(a) and 2(b)). The highest number of SPION@CMD and SPION@CMD@triptorelin complexes had a diameter of 160 nm and 116 nm, respectively. This phenomenon indicates narrow particle size distribution and monomodal population of the particles due in part to the contribution of triptorelin with SPIONs. Zeta potential was measured at ∼−72.4 mV for SPION@CMD and ∼−31.5 mV for SPION@CMD@triptorelin (Figures 2(c) and 2(d)).

**Figure 3:**
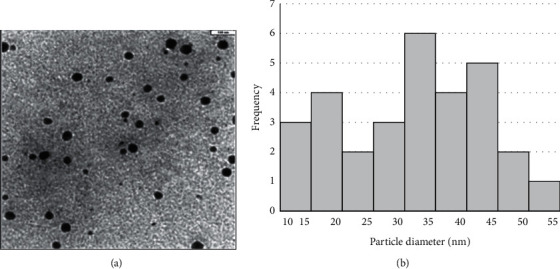
(a) TEM micrograph of SPIONs. (b) Size distribution histogram of SPIONs.

According to the TEM image, the synthesized SPION@CMD showed a spherical and homogeneous morphology (Figure 3(a)). The particle size distribution histogram obtained from TEM image (Figure 3(b)) revealed that the largest number of SPION@CMD nanoparticles had dimensions between 30 and 45 nm with an average size of 31.35 ± 11.1 nm.

**Figure 4:**
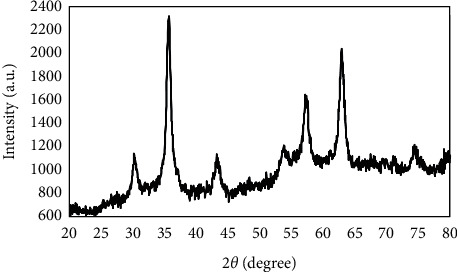
XRD pattern of sample SPIONs synthesized by a coprecipitation method. The XRD system acted at 40 kV and 30 mA in a 2*θ* range of 20°–80°.

Figure 4 shows the obtained XRD data of SPIONs. The XRD profile revealed that maximum XRD peak occurred at 2*θ* value of 35.7° that represented a typical SPION with an interlayer spacing value of 3.83242 A. The position and relative intensity of all peaks were matched with standard magnetite Fe_3_O_4_ pattern (JCPDS card, file no. 19–0629), indicating that the synthesized nanoparticles are magnetic (Fe_3_O_4_) crystals. The peaks of 220, 311, 400, 422, 511, and 440 were the main peaks of the crystalline pattern of hydrophobic SPIONs. The mean size of crystals (*D*) was estimated based on the Sherrer equation:

(2) $$\begin{array}{c} D=\frac{K\lambda }{\beta \;\cos\;\theta }, \end{array}$$

where *K* is the Scherrer constant (0.9), *λ* is the wavelength (0.1542 nm), *β* is the FWHM (in radians), and *θ* is the peak angular position. The peaks of 220, 311, 400, 422, 511, and 440 were the main peaks of the crystalline pattern of hydrophobic SPIONs. The size of SPIONs crystal was calculated by the most intensive peak (311) with a value of ∼7.95 nm.

**Figure 5:**
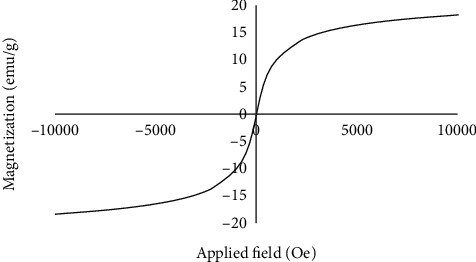
Magnetic hysteresis curve for SPIONs at the magnetic field in the range of 0 to 10 kOe at 27°C.

The magnetic characteristics of synthesized SPIONs were evaluated by VSM.  Figure 5 shows that nanoparticles possessed superparamagnetism at 27°C with a saturation magnetization (Ms) value of 18.26 emu/g.

**Figure 6:**
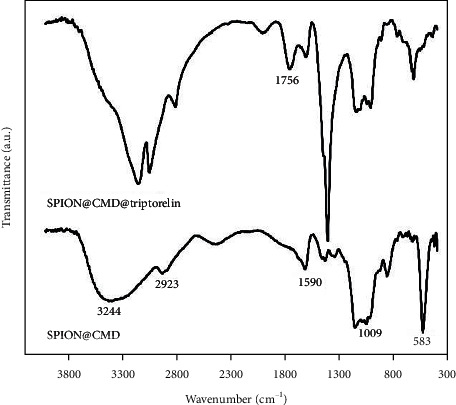
FTIR spectra of Fe_3_O_4_ and SPIONs coated with triptorelin.

Figure 6 shows FTIR of SPION@CMD and SPION@CMD@triptorelin. In the spectrum of SPION@CMD, the band at 3244 cm^−1^ was corresponded to the stretching vibration and bending vibration of the O-H bonds related to the adsorbed water groups. The peak at 583 cm^−1^ was related to Fe-O bonds. The peaks at 2923 cm^−1^ and 583 cm^−1^ were related to the CH2 and C-O bonds of carboxymethylated dextran (CMD), respectively. Also, the peak at 1590 cm^−1^ corresponds to the carbonyl of the asymmetric carboxyl group, and the bond displacement is due to the complexation of the carbonyl group with the surface of SPIONs.

In the spectrum of SPIONs coated with triptorelin, the peak at 1756 cm^−1^ was attributed to the existence of the *γ*-lactam and is evidence of peptide binding on the surface of SPIONs. Therefore, the FTIR spectra results confirmed the surface modification of magnetite nanoparticles by triptorelin.

### 3.2. Cell Viability Assay of the Synthesized Formulations

**Figure 7:**
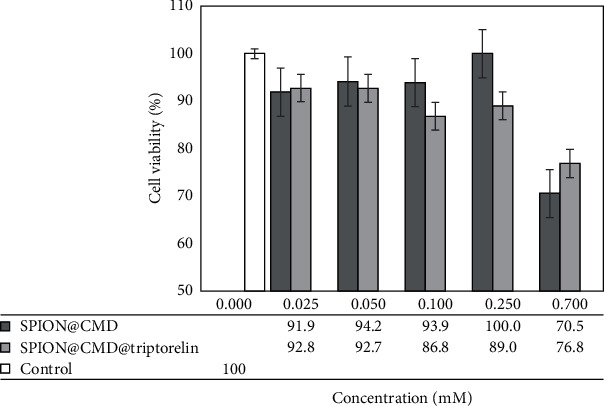
In vitro cytotoxicity of SPION@triptorelin tested on MDA-MB-231 cell line after 24 h incubation at 37°C. Data are shown as mean ± SD (*n* = 5).

The cytotoxicity of SPION@CMD and SPION@CMD@triptorelin was evaluated by MTT assay. The obtained data illustrated that the difference between cytotoxicity of SPION@CMD at concentrations of 0.025, 0.05, 0.1, 0.25, and 0.7 mM and control group was not significant (^*∗*^*p* > 0.05), while 0.7 mM SPION@CMD showed a significant difference in comparison with the control group. The test also showed a survival rate of more than 60% for the maximum concentration of 0.7 mM SPION@CMD@triptorelin (Figure 7). So, the MTT assays confirmed the lack of cellular cytotoxicity of SPION@CMD@triptorelin formulation.

### 3.3. Cellular Uptake Level of Synthesized Formulations

**Figure 8:**
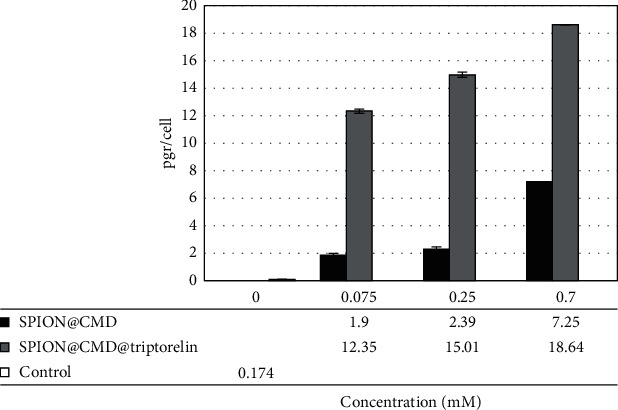
Cellular uptake level of synthesized formulations.

According to the atomic absorption spectroscopy results, the intracellular iron of MDA-MB-231 cells treating with triptorelin-coated SPIONs was 6.50, 6.28, and 2.57 more than noncoated SPIONs at 0.075, 0.25, and 0.7 mM concentrations, respectively (Figure 8). It is confirmed that the cellular uptake efficiency of targeted SPION@CMD@triptorelin was better than SPION@CMD.

**Figure 9:**
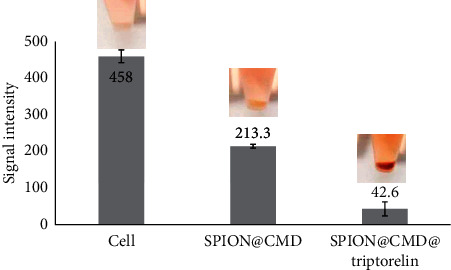
Signal intensity depended on the nanostructures (SPION@CMD and SPION@CMD@peptide). *T*_2_-weighted MR image in cellular medium with 24-hour incubation time. Cell played a role as a control sample.

The ability of the synthesized nanoparticles to targeting specifically to the MDA-MB-231 cells was also confirmed with MR imaging techniques. The results displayed in Figure 9 demonstrate that nanoparticles functionalized with triptorelin reduced by more than 90% MR image intensity compared with the 53% reduction in SPION@CMD at a Fe concentration of 0.25 mM.

### 3.4. MRI Relaxometry

Relaxometry refers to the measurement of power of nanoparticles in MRI as contrast agent. To determine the specific magnetic properties of nanoparticles, the solutions of SPION@CMD and SPION@CMD@triptorelin were prepared in water at 0.15, 0.3, 0.9, 1.2, 2.4, and 3 mM Fe for relaxivity measurements.

**Figure 10:**
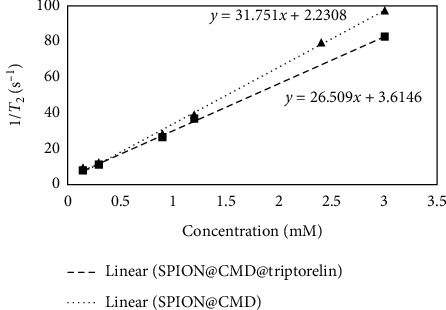
Calculated *T*_2_ relaxation rate and relaxivity at various Fe concentrations of SPION@CMD and SPION@CMD@triptorelin. Spin echo multisection pulse sequence with fix TR of 2000 ms and different TEs ranging between 13.8 and 220.8 ms for *T*_2_ measurement at various concentrations (0.15–0.3 mM) that were prepared in the deionized water, and deionized water played a role as a control sample.

The *r*_2_ relativity was calculated as 31.75 mM^−1^s^−1^ according to the linear plot slope of the SPION@CMD concentration depending on the inverse *T*_2_ with *R*^2^ = 0.9965, while the *r*_2_ relativity was obtained as 26.51 mM^−1^·s^−1^ with *R*^2^ = 0.9991 for SPION@CMD@triptorelin which was lower than SPION@CMD (Figure 10)

**Figure 11:**
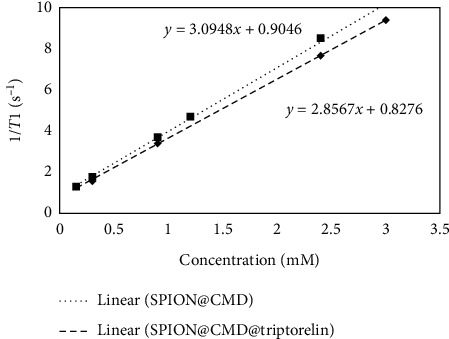
Calculated *T*_1_ relaxation rate and relaxivity at various Fe concentrations of SPION@CMD and SPION@CMD@triptorelin. *T*_1_-weighted images were acquired with fix TE of 8.7 ms and TRs ranging between 100 and 2000 ms for *T*_1_ measurement at various concentrations (0.15–0.3 mM) that were prepared in the deionized water, and deionized water played a role as a control sample.

The *r*_1_ parameter for SPION@CMD and SPION@CMD@triptorelin was calculated as 3.10 mM^−1^·s^−1^ and 2.86 mM^−1^·s^−1^, respectively (Figure 11). The *r*_2_/*r*_1_ ratio is an interesting sensitive parameter that is used to identify the category of the contrast agents (*T*_1_ or *T*_2_ contrast agent). The *r*_2_/*r*_1_ ratio was calculated as 10.24 and 9.27 for SPION@CMD and SPION@CMD@triptorelin, respectively.

## 4. Discussion

In modern medicine, the use of targeted imaging probes is very important to improve diagnostic methods. One of the features of an imaging probe for clinical use is stability and biocompatibility. The aggregation and the instability are the main drawbacks of using SPIONs at physiological pH. To overcome these limitations, various surface coatings have been used to modify their surface properties [22]. It was also demonstrated that modification of SPIONs with carboxyl (-COOH) groups provide appropriate sites can be conjugated with any drugs and/or natural compounds containing amine (-NH_2_) group or their combinations [23, 24].

On the other hand, although contrast agents improve the quality of the images, the injectable dose of them is limited. The utilization of ligands such as peptides can specifically increase the cell uptake by targeted cells, which results in applying lower concentration of the contrast agent. Using this approach, we used carboxymethylated dextran which contributes to the SPIONs with triptorelin peptide. An insight into the possibility of SPION@CMD@triptorelin formation was also confirmed by the FTIR. The coating of SPIONs can be also attributed to the reduced shielding effect of Fe_3_O_4_ corona which can facilitate the implementation of SPIONs as contrast agents for MRI. The protein corona formation is interaction of bare Fe_3_O_4_ nanoparticles with the plasma proteins [25]. Recent studies have found that bare Fe_3_O_4_ nanoparticles could induce the cytotoxicity and apoptosis by ROS generation and oxidative stress [26, 27]. The coating of Fe_3_O_4_ could reduce the cytotoxicity of these nanoparticles, whenever MTT assays confirmed the lack of cellular cytotoxicity of SPION@CMD@triptorelin.

The evaluation of cellular uptake of formulations on MDA-MB-231 cell line indicated the importance of triptorelin peptide on the uptake of the targeted formulation. With increasing concentration, cellular uptake of nanoparticles, either targeted or nontargeted ones, increases. In all concentrations, the cellular uptake for SPION@CMD@triptorelin was higher than SPION@CMD. This is in agreement with previous studies in which delivery of MRI agents with peptides increased the cellular internalization of functionalized-SPIONs in vitro [28]. The mechanism of triptorelin-based internalization gives rise to receptor-mediated endocytosis [17], resulting in efficient internalization of formulation into the GnRH-expressing cells.

In Poller et al.'s study, cellular uptake of nontargeted SPIONs for different breast cell lines was investigated, which at 50 µg/ml concentration, the cellular uptake of 0.3 pg/cell was reported for MDA-MB-231 [29]. However, we obtained cellular uptake of 7.25 pg/cell at the concentration of 0.7 mM (39.2 *μ*g/ml) for the same cell line and incubation time [30].

The size of synthesized Fe_3_O_4_-based complexes influences the magnetic properties and internalization of iron oxide nanoparticle into the target cell [31]. In our research, the obtained average hydrodynamic diameters for SPION@CMD and SPION@CMD@triptorelin by DLS was 160 and 116 nm, respectively. The TEM image showed that the synthesized SPIONs had uniform and heterogeneous morphology with an average size of 31 nm, while the size distribution histogram obtained from TEM image revealed also that the SPIONs as low as 10–20 nm. The small sizes of SPION@CMD in TEM image in comparison to DLS might be the determination of the hydrodynamic diameter of the CMD-coated SPIONs via DLS in aqueous solution incorporating surface-bound water layers in their measurement, while TEM estimates the actual core size of dried SPIONs. However, the larger size in TEM image might be emanated from higher aggregation tendency of the smaller SPIONs due to Van der Waals forces between the particles [32]. The small size and modified surface area of SPIONs leading to superparamagnetic behavior [33, 34]. Through the analysis of XRD pattern, the crystal size of the synthesized SPION@CMD was assessed by the Sherrer equation with a small size of 7.95 nm. Such small size could be related to the size of single crystal while TEM images revealed the particle dimension. However, slightly higher sizes could be optimal for the enhancement of the *r*_2_ relaxivity [35, 36]. In addition, the small-sized SPIONs increased the surface-to-volume ratio, and thus the increased dead layer component decreased magnetization (*M*_*S*_) [37].

MR images (*T*_2_-weighted imaging) of SPION@CMD and SPION@CMD@triptorelin in an aqueous environment evaluated a noticeable contrast by changing the concentrations of SPIONs. Therefore, SPION@CMD@triptorelin could be considered as a targeted negative contrast agent. The *r*_2_ value of SPION@CMD and SPION@CMD@triptorelin was estimated at 31.75 mM^−1^·s^−1^ and 26.50 mM^−1^·s^−1^, respectively, by a magnetic relaxometry at a 1.5 T conventional MRI system. The size, mass magnetization (*M*_*S*_), and the magnetic field strength are factors affect the *r*_2_ value [38, 39]. In agreement with previous studies, the obtained *r*_2_ indicated that *T*_2_ relativity also depends on the concentration of SPIONs. It was also demonstrated that the large sizes of SPIONs and stronger magnetic field lead to the higher *r*_2_/*r*_1_. The high value of *r*_2_/*r*_1_ ratio indicated *T*_2_ contrast agent [40, 41]. In the present study, the calculated *r*_2_/*r*_1_ indicated SPION@CMD@triptorelin as a good candidate for a negative contrast agent in clinical magnetic field strength.

## 5. Conclusion

In the present study, SPION@CMD@triptorelin, as a targeted probe in GnRH + cancer cells, was successfully synthesized by a coprecipitation method and the average size of 31 nm. *T*_2_ relaxation times of hydrogen protons in aqueous solutions of varying concentrations were determined with a conventional MRI. *T*_2_ relaxivities (*r*_2_) were determined to be 31.75 mM^−1^·s^−1^ and 26.50 mM^−1^·s^−1^ for SPION@CMD and SPION@CMD@triptorelin, respectively. Moreover, *r*_2_/*r*_1_ ratio of the targeted SPIONs was calculated as 10.26; this finding demonstrated the potential use of the synthesized SPION@CMD@triptorelin as appropriate targeted negative contrast agents at conventional MRI system at low applied concentration. In addition, in vitro cell viability assays indicate that the SPION@CMD@triptorelin showed no cellular viability reduction for concentrations up to 0.7 mM. Our findings suggested that SPION@CMD@triptorelin can be used in the future as a targeted theranostic agent for improving diagnostic and therapeutic application. It can be concluded that SPION@CMD@triptorelin loaded with anticancer drug provides a theranostic platform for specific delivery of MRI agents and drugs.

## Data Availability

The data used to support the findings of this study are included within the article.
